# Severe Obesity and Cardiometabolic Risk in Children: Comparison from Two International Classification Systems

**DOI:** 10.1371/journal.pone.0083793

**Published:** 2013-12-27

**Authors:** Giuliana Valerio, Claudio Maffeis, Antonio Balsamo, Emanuele Miraglia Del Giudice, Claudia Brufani, Graziano Grugni, Maria Rosaria Licenziati, Paolo Brambilla, Melania Manco, on the behalf of the Childhood Obesity Group of the Italian Society of Pediatric Endocrinology and Diabetology

**Affiliations:** 1 Dipartimento di Scienze Motorie e del Benessere, Università degli Studi di Napoli Parthenope, Napoli, Italy; 2 Unità Operativa Complessa di Diabetologia, Nutrizione Clinica e Obesità in Età Pediatrica, Università di Verona, Verona, Italy; 3 Dipartimento di Scienze Mediche e Chirurgiche, Programma di Endocrinologia Pediatrica, Unità Operativa di Pediatria, Università di Bologna, Policlinico S. Orsola-Malpighi, Bologna, Italy; 4 Dipartimento della Donna, del Bambino e di Chirurgia Generale e Specialistica, Seconda Università di Napoli, Napoli, Italy; 5 Unità Operativa Complessa di Endocrinologia e Diabetologia, Ospedale Pediatrico Bambino Gesù, Roma, Italy; 6 Divisione di Auxologia, Istituto Auxologico Italiano, Piancavallo, Verbania, Italy; 7 Unità Operativa Complessa di Auxologia ed Endocrinologia - Dipartimento di Pediatria Sistematica e Specialistica - Azienda Ospedaliera di Rilevanza Nazionale Santobono-Pausilipon-Annunziata, Napoli, Italy; 8 Dipartimento di Pediatria, Istituto Scientifico San Raffaele, Milano and Azienda Sanitaria Locale 2, Melegnano, Milano, Italy; 9 Direzione Scientifica, Area di Ricerca Malattie Multifattoriali, Obesità e Diabete, Ospedale Pediatrico Bambino Gesù, Roma, Italy; Old Dominion University, United States of America

## Abstract

**Objectives:**

There is no agreed-upon definition for severe obesity (Sev-OB) in children. We compared estimates of Sev-OB as defined by different cut-points of body mass index (BMI) from the Centers for Disease Control and Prevention (CDC) or the World Health Organization (WHO) curves and the ability of each set of cut-points to screen for the presence of cardiometabolic risk factors.

**Research Design and Methods:**

Cross-sectional, multicenter study involving 3,340 overweight/obese young subjects. Sev-OB was defined as BMI ≥99^th^ percentile or ≥1.2 times the 95^th^ percentile of the CDC or the WHO curves. High blood pressure, hypertriglyceridemia, low High Density Lipoprotein -cholesterol and impaired fasting glucose were considered as cardiometabolic risk factors.

**Results:**

The estimated prevalence of Sev-OB varied widely between the two reference systems. Either using the cut-point ≥99^th^ percentile or ≥1.2 times the 95^th^ percentile, less children were defined as Sev-OB by CDC than WHO (46.8 vs. 89.5%, and 63.3 vs. 80.4%, respectively p<0.001). The CDC 99^th^ percentile had lower sensitivity (58.5 vs 94.2), higher specificity (57.6 vs 12.3) and higher positive predictive value (34.4 vs 28.9) than WHO in identifying obese children with ≥**2** cardiometabolic risk factors. These differences were mitigated using the 1.2 times the 95^th^ percentile (sensitivity 73.9 vs. 88.1; specificity 40.7 vs. 22.5; positive predictive value 32.1 vs. 30.1). Substantial agreement between growth curves was found using the 1.2 times the 95^th^ percentile, in particular in children ≤10 years.

**Conclusions:**

Estimates of Sev-OB and cardiometabolic risk as defined by different cut-points of BMI are influenced from the reference systems used. The 1.2 times the 95^th^ percentile of BMI of either CDC or WHO standard has a discriminatory advantage over the 99^th^ percentile for identifying severely obese children at increased cardiometabolic risk, particularly under 10 years of age.

## Introduction

Childhood obesity is a major public health threat [1], [2]. Estimates of its prevalence rely on the definition criteria used, with clear implications on surveillance and clinical practice. Body mass index (BMI) was recommended as a screening rather than a diagnostic tool for pediatric obesity [3], [4]. Indeed, BMI correlates with total body fat and cardiometabolic risk factors [5]. Higher BMI among children is associated with higher levels of blood pressure, lipids and other factors that in adults are related to cardiovascular disease risk [5], but the implications of a given level of BMI for children's future cardiometabolic health are unclear [6].

Definitions of pediatric obesity are statistical rather than risk based [6]. Current definitions are based on arbitrarily chosen cut-points of BMI percentiles [7]. Since the extent of overweight is increased [8], there is strong interest in identifying children with severe obesity (Sev-OB) in order to identify children who may deserve intensive treatment. There is still no uniform consensus on definition of Sev-OB in children. Methods based upon percentiles are widely used in clinical practice, and easier for patients and families to understand. Experts suggested that Sev-OB be defined as BMI ≥99^th^ percentile [4] or BMI ≥1.2 times the 95^th^ percentile [9], this latter corresponds to the definition of Sev-OB in adults (class 2, BMI ≥35 or approximately 1.2 times the BMI 30 cut point). Nowadays different BMI standards are available, the most employed being those from the Centers for Disease Control and Prevention (CDC) [10] or the World Health Organization (WHO) [11]. Values of BMI at the 99^th^ percentile specific for gender and age are available from both the CDC and the WHO systems: the former were extrapolated from the CDC-supplied lambda-mu-sigma (LMS) values estimated on nationally representative growth charts derived from the 1999–2004 National Health and Nutrition Examination Survey (NHANES) [5]; the latter were provided from the newly statistically reconstructed curves that used the core sample of the original National Center for Health Statistics charts (NHES II and III and NHANES I) when obesity was not yet epidemic [11].

Studies evaluating associations between health outcomes and the excess weight categories defined by different sets of cut-points are needed to inform the decision on which method best assesses risk.

As the use of different methods is expected to give different estimates of Sev-OB, our aim was to assess the extent to which different cut-points of BMI by either CDC or WHO can affect the estimation of Sev-OB in children, but prominently to assess the ability of each set of cut-point to screen children with Sev-OB and clustered cardiometabolic risk.

## Methods

Between January 2005-December 2009 fifteen pediatric obesity services participated in this cross-sectional study, providing medical records of 3,802 overweight/obese subjects. Subjects were selected randomly in numbers weighted to each pediatric service population. Inclusion criteria were: Caucasian race, age (5–18 years), having complete data set. Exclusion criteria were: secondary obesity, chronic diseases, malformations and chronic use of drugs leading to metabolic disturbances (such as steroids). Three-thousand-three-hundred-forty overweight/obese subjects (1703 males, 1637 females) were effectively included in the study; they were geographically distributed across the northern (n = 908, 27.2%), central (n = 825, 24.7%), and southern (n = 1607, 48.1%) Italian regions. One-hundred-twenty-two subjects (50 males, 72 females) were excluded because they did not fulfill the required age criteria. Three-hundred-forty subjects (172 males, 168 females) were excluded because of missing values; their baseline characteristics (sex, age, BMI) did not differ from the study population (data not shown). Anonymized data are available upon request.

### Ethics Statement

The study protocol was approved by the Childhood Obesity Group Review Board of the Italian Society of Pediatric Endocrinology and Diabetology. Neither approval from Ethics Committee nor individual written consent by parents and adolescents were requested according to the Italian law (anonymous aggregated data) (legislative decree June, 30^th^ 2003, issue 196, attachment 4 and “Autorizzazione generale al trattamento dei dati personali effettuato per scopi di ricerca scientifica” - March 1^st^, 2012 (Gazzetta Ufficiale issue 72, March 26^th^, 2012; www.garanteprivacy.it). The study protocol conformed to the guidelines of the European Convention of Human Rights and Biomedicine for Research in Children. All measures were taken to ensure the confidentiality of families and children whose data were used. Personal and clinical data of patients were rendered anonymus before transmission and analysis. The directive 95/46/EC of the European Parliament and of the Council of 24 October 1995 on the protection of personal data was complied with for data storage and handling in order to ensure patient data protection and confidentiality.

### Anthropometry and Blood Pressure Evaluation

Body weight was determined to the nearest 0.1 kg on accurate and properly calibrated standard beam scales, in minimal underclothes and no shoes. Height was measured to the nearest 0.5 cm on standardized, wall-mounted height boards according to standardized procedures. In brief, the subject stood straight, with feet placed together and flat on the ground, heels, buttocks and scapulae against the vertical backboard, arms loose and relaxed with the palms facing medially. The head was carefully positioned in the Frankfurt plane, i.e. with the lower margins of the orbit in the same horizontal plane as the upper margin of external auditory meatus [12]. The BMI was calculated as weight divided by square of height (kg/m^2^). Height and weight were measured in each centre by the same investigator, who was specifically trained in anthropometry; the average of the two closest measurements of height was used for the analysis; if a difference of 0.5 cm or more was found, a third measurement was taken.

Blood pressure (BP) was measured using a mercury sphygmomanometer, according to a standardized protocol [13]. Briefly, the cuffs had bladders long enough to encircle at least one-half of the upper arm without overlapping and widths that covered at least two-thirds of the upper arm. The average of three BP values was used for analysis.

### Biochemical Parameters

Fasting venipuncture samples were drawn for plasma triglycerides, high-density lipoprotein (HDL)-cholesterol and glucose measurements and analyzed with standard techniques: triglycerides were measured enzymatically, the HDL-cholesterol fraction was obtained after precipitation using a phosphotungistic reagent, glucose was measured using a glucose oxidase method. Although analyses were performed in different laboratories, all centres belong to the Italian National Health system and are certified according to International Standards ISO 9000 (www.iso9000.it/), undergoing to semi-annual quality controls and inter-lab comparisons; this contributes to limit the potential differences among laboratories.

### Case definitions

Overweight (OW), moderate obesity (Mod-OB), and Sev-OB were defined using the CDC or the WHO standards. OW was defined as a BMI-for-age value ≥85^th^ and <95^th^ percentile, Mod-OB as a value ≥95^th^ and <99^th^ percentile, Sev-OB as a value ≥99^th^ percentile [4] or ≥1.2 times the 95^th^ percentile [9]. The 85^th^ and 95^th^ cut-points by CDC were retrieved from Kuczmarski RJ *et al*. [10], while the CDC 99^th^ values were applied from an expert committee recommendations [4]. The 85^th^, 95^th^ and 99^th^ cut-points by WHO were retrieved from tables available at http://www.who.int/childgrowth/standards/bmi_for_age/en/index.html) [8].

The following cardiometabolic risk factors were assessed: high blood pressure (systolic and/or diastolic BP ≥95^th^ percentile for age, sex and height); hypertriglyceridemia (triglycerides >95^th^ percentile for age and sex), low levels of HDL-cholesterol (HDL-cholesterol <5^th^ percentile for age and sex; impaired fasting glucose (glucose ≥5.6 mmol/L) [13], [14]. Clustering of risk was defined in those subjects having obesity and two or more cardiometabolic risk factors.

### Statistical analysis

The confidence interval estimation performed to determine the sample size indicated that a size of 375 produced a 95% confidence interval equal to the sample proportion plus or minus 0.05 when the estimated proportion was 0.42 (according to a recent estimated incidence of 42% of adverse cardiometabolic factors in Italian overweight/obese children [15].

Continuous data are reported as means and standard deviations (SD), with categorical data as counts and percentages. Variables not normally distributed (weight, BMI, triglycerides, HDL-cholesterol) were logarithmically transformed; for clarity of interpretation, results are expressed as untransformed values. Intergroup comparisons were made by the Student's t-test.

The diagnostic accuracy of either BMI ≥99^th^ percentile or BMI ≥1.2 times the 95^th^ percentile to discriminate the presence of single or clustered cardiometabolic risk factors (≥2 risk factors) was assessed. Receiver operating characteristic (ROC) curve analysis was used to evaluate accuracy, specificity and sensitivity of both WHO and CDC BMI ≥99^th^ percentile or BMI ≥1.2 times the 95^th^ percentile to identify patients with high blood pressure, dyslipidemia (high triglycerides or low HDL cholesterol), impaired fasting glucose and clustered risk factors. Accuracy is measured by the area under the ROC curve (AUROC) with an area of 1 representing a perfect test and an area of 0.5 a worthless test. Hence, we assessed the: 1) positive predictive value (PPV) (proportion of Sev-OB children who have single or clustered cardiometabolic risk factors), 2) negative predictive value (NPV) (proportion of Sev-OB children who do not have single or clustered cardiometabolic risk factors), 3) sensitivity (proportion of children with single or clustered cardiometabolic risk factors who are Sev-OB), 4) specificity (proportion of children without single or clustered risk factors who are not Sev-OB). Since these statistics, such as PPV and PPN, depend on the prevalence of disease factors, we analyzed also the positive likelihood ratio (LR+) and the negative likelihood ratio (LR−) [16]. The LR+ is the probability of being Sev-OB among children who have adverse risk factor levels (sensitivity) divided by the probability of being Sev-OB among children who have normal risk factor levels (1 – specificity). The LR− is the probability of not being Sev-OB among children who have a cardiometabolic risk factor level (1 – sensitivity) divided by the probability of not being Sev-OB among children who have normal risk factor levels (specificity). In general, a better test will have a higher LR+ and a lower LR− than an inferior test [17].

We used kappa statistics to determine the level of agreement between CDC and WHO growth curves in identifying single or clustered cardiometabolic risk factors. Kappa coefficients were interpreted as follows: <0 less than chance agreement; 0.01–0.20 slight agreement; 0.21–0.40 fair; 0.41–0.60 moderate; 0.61–0.80 substantial; 0.81–1.00 almost perfect agreement. The Statistical Package of Social Sciences (SPSS, Chicago, IL, USA) for Windows software program release 18.0 was used. A p value <0.05 was considered significant.

## Results

The anthropometric characteristics and cardiometabolic profile of the total study population and of groups stratified by gender and age have been presented in **Table 1**.

**Table 1:** Anthropometric characteristics and cardiometabolic profile of the total study population and of groups stratified by gender and age.

	TOTAL	GENDER		AGE CLASS	
		Males	Females	≤10 yrs	≥11 yrs
**Males/Females (n)**	1703/1637	1703	1637	543/582≠	1160/1055
**Age (years)**	11.2±2.9	11.2±2.7	11.2±3.0	8.0±1.2	12.8±1.9
**Height (cm)**	148.7±14.8	150.1±15.2	147.2±14.2	133.9±9.6	156.2±10.8
**Weight (kg)**	69.4±23.2	70.7±23.7	68.1±22.6	49.8±11.7	79.3±21.1
**BMI (kg/m^2^)**	30.5±5.7	30.6±5.5	30.5±5.9	27.5±3.9	32.1±5.8
**Excess BMI category, no (%)**					
**OW_CDC_ (≥85^th^ BMI < 95^th^ CDC)**	140 (4.2)	46 (2.7)	94 (5.7)	20 (1.8)	120 (5.4)
**OW_WHO_ (≥85^th^ BMI < 95^th^ WHO)**	35 (1.0)	12 (0.7)	23 (1.4)	5 (0.4)	30 (1.4)
**Mod-OB_CDC_ (≥95^th^ BMI <99^th^ CDC)**	1637 (49.0)	775 (45.5)	862 (52.7)	369 (32.8)	1268 (57.2)
**Mod-OB_WHO_ (≥ 95^th^ BMI < 99^th^ WHO)**	316 (9.5)	112 (6.6)	204 (12.5)	38 (3.4)	278 (12.6)
**Sev-OB_CDC_ (≥ 99^th^ BMI CDC)**	1563 (46.8)	882 (51.8)**	681 (41.6)	736 (65.4)^§^	827 (37.3)
**Sev-OB_WHO_ (≥ 99^th^ BMI WHO)**	2989 (89.5)	1579 (92.7)**	1410 (86.1)	1082 (96.2)^§^	1907 (86.1)
**BMI ≥ 1.2 times the 95^th^ BMI CDC**	2115 (63.3)	1173 (68.9)**	942 (57.5)	852 (75.7)^§^	1263 (57.0)
**BMI ≥ 1.2 times the 95^th^ BMI WHO**	2687 (80.4)	1455 (85.4)**	1232 (75.3)	987 (87.7)^§^	1700 (76.7)
**Cardiometabolic abnormality, n (%)**					
**SBP/DBP** ≥**95^th^ percentile**	1143 (34.2)	580 (34.1)	563 (34.4)	334 (29.7)^§^	809 (36.5)
**TG >95^th^ percentile**	1146 (34.3)	579 (34.0)	567 (34.6)	555 (49.3)^§^	591 (26.7)
**HDL-cholesterol <5^th^ percentile**	951 (28.5)	562 (33.0)**	389 (23.8)	229 (20.4)^§^	722 (32.6)
**Glucose ≥5.6 mmol/L**	115 (3.4)	63 (3.7)	52 (3.2)	28 (2.5)#	87 (3.9)
**Clustered risk factors, no (%)**					
**0 cardiometabolic abnormality**	1105 (33.1)	539 (31.7)*	566 (34.6)	357 (31.7)≠	748 (33.8)
**1 cardiometabolic abnormality**	1317 (39.4)	661 (38.8)	656 (40.1)	445 (39.6)	872 (39.4)
**2 cardiometabolic abnormalities**	726 (21.7)	393 (23.1)	333 (20.3)	271 (24.1)	455 (20.5)
**≥3 cardiometabolic abnormalities**	192 (5.7)	110 (6.5)	82 (5.0)	52 (4.6)	140 (6.3)

Comparison between genders * p<0.04; **p<0.001.

Comparison between age class # p<0.04; ≠p<0.03; ^§^p<0.001.

BMI, Body Mass Index; OW, overweight; Mod-OB, Moderate Obesity; Sev-OB, Severe Obesity; CDC, Centers for Disease Control and Prevention; WHO, World Health Organization; SBP, systolic blood pressure; DBP, diastolic blood pressure; TG, triglycerides.

Distribution of subjects among the different categories of excess weight was significantly different between CDC and WHO (**Table 1**). The frequency of Sev-OB was significantly higher in boys than girls and in younger than older children (p<0.001), independently of the classification system. Either using the cut-point ≥99^th^ percentile or ≥1.2 times the 95^th^ percentile, less children in the total sample were defined as Sev-OB by CDC than WHO (46.8 vs. 89.5%, and 63.3 vs. 80.4%, respectively p<0.001). Within the CDC system, the 99^th^ percentile provided lower estimates of Sev-OB than the 1.2 times the 95^th^ percentile (46.8 vs. 63.3%, p<0.001); within the WHO system, the 99^th^ percentile provided slightly higher, but significant estimates than the 1.2 times the 95^th^ percentile (89.5 vs. 80.4%, p<0.001). Similar differences (p<0.001) were found in groups stratified by gender or age class.

There were few sex differences in the frequency of cardiometabolic risk factors: more boys than girls had low HDL-Cholesterol levels or clustering of at least 2 cardiometabolic abnormalities. Older children exhibited higher frequency of all the cardiometabolic abnormalities, apart hypertriglyceridemia, than younger children (Table 1).

**Table 2** shows the anthropometric and laboratory characteristics of subjects classified as Sev-OB. Using the CDC 99^th^ percentile, subjects with Sev-OB were more frequently male and younger than the similar category defined by WHO; they also had significantly higher BMI, systolic and diastolic BP and lower HDL-cholesterol. Using the CDC 1.2 times the 95^th^ percentile, subjects with Sev-OB had higher weight and BMI than the similar category defined by WHO.

**Table 2:** Characteristics of population with severe obesity as defined according to BMI-for-age ≥99^th^ percentile or ≥1.2 times the 95^th^ percentile, by CDC or WHO.

	99^th^ percentile			1.2 times the 95^th^ percentile		
	CDC	WHO	*P*	CDC	WHO	*P*
**Number**	1563	2989		2115	2687	
**Males n (%)**	882 (56.4)	1579 (52.8)	.021	1173 (55.5)	1455 (54.1)	.365
**Age (years)**	10.5±3.1	11.0±2.9	<.001	10.8±2.9	10.9±2.9	.236
**Height (cm)**	146.4±16.5	148.2±14.9	<.001	147.8±15.5	148.2±14.8	.363
**Weight (kg)**	74.9±27.9	70.4±23.8	<.001	73.8±25.4	71.6±23.9	<.009
**BMI (kg/m^2^)**	33.6±6.2	31.1±5.7	<.001	32.7±5.8	31.7±5.6	<.001
**Waist (cm)**	96.8±16.9	93.0±15.3	<.001	95.7±15.8	94.0±15.3	<.01
**Systolic BP (mmHg)**	116.9±15.3	115.8±14.5	<.02	116.7±14.8	116.1±14.4	.224
**Diastolic BP (mmHg)**	70.4±11.1	69.6±10.7	<0.03	70.4±10.9	69.9±10.7	.110
**Triglycerides (mg/dl)**	1.07±0.54	1.05±0.53	.065	1.07±0.53	1.06±0.53	.358
**HDL-Cholesterol (mg/dl)**	1.18±0.31	1.20±0.30	<.006	1.18±0.31	1.19±0.30	.196
**Glucose (mg/dl)**	4.7±0.5	4.6±0.5	.726	4.6±0.5	4.6±0.5	.995

CDC, Centers for Disease Control and Prevention; WHO, World Health Organization; BMI, Body mass Index; BP, Blood Pressure; HDL, High Density Lipoprotein.

**Figure 1:**
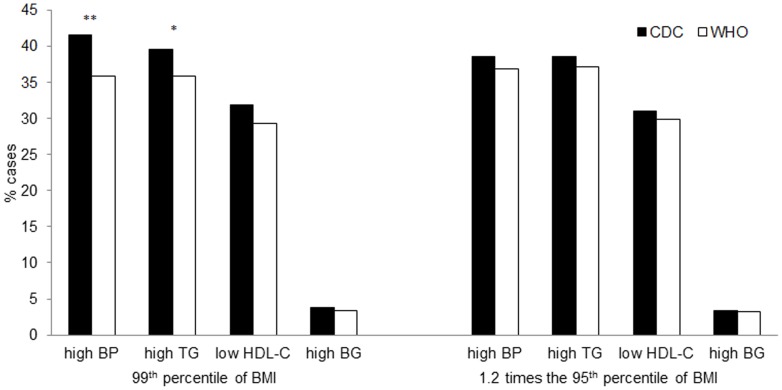
Relative frequency of single cardiometabolic risk factors in children with severe obesity defined according to BMI-for-age ≥99^th^ percentile or ≥1.2 times the 95^th^ percentile, by CDC or WHO.

An examination of the individual cardiometabolic risk factors indicated that for each of the four risk factors the CDC 99^th^ percentile had lower sensitivity and NPV, while it showed higher specificity and PPV than WHO **(** **Table 3** **)**. Similar results were found in samples stratified by gender or age (data not shown). The discrepancies in sensitivity and specificity between CDC and WHO were strongly reduced using the 1.2 times the 95^th^ percentile **(** **Table 4** **)**. Using the BMI 99^th^ percentile the level of agreement between CDC and WHO growth curves in identifying single cardiometabolic risk factors was slight (kappa coefficients ranged from 0.03 to 0.16 for all risk factors). On the contrary, using the 1.2 times the 95^th^ percentile, kappa coefficients were greater than 0.60 for high blood pressure, high TG levels and low HDL-cholesterol levels, suggesting substantial agreement between CDC and WHO growth curves. The ability of 99^th^ percentile and 1.2 times the 95^th^ percentile of BMI to identify subjects with single cardiometabolic risk factors is shown in **Figure 1**. Using the 1.2 times the 95^th^ percentile no difference between CDC and WHO was found in any of the cardiometabolic risk factors, while a significant difference regarding high blood pressure and hyper-triglyceridemia (p<0.0002 and p<0.02, respectively) was found using the 99^th^ percentile.

**Table 3:** AUROC, sensitivity, specificity, PPV, NPV, LR^+^, LR^−^ and kappa of categories of severe obesity defined as BMI ≥99^th^ percentile by CDC or WHO for predicting individual cardiometabolic risk.

	Severe obesity defined as BMI ≥99^th^ percentile														
	AUROC (95%CI)		Sensitivity		Specificity		PPV		NPV		LR^+^		LR^−^		kappa
	CDC	WHO	CDC	WHO	CDC	WHO	CDC	WHO	CDC	WHO	CDC	WHO	CDC	WHO	
**SBP/DBP ≥95^th^ percentile**	0.59 (0.58–0.62)	0.59 (0.57–0.61)	56.9	93.8	58.5	12.7	41.6	35.9	72.3	79.8	1.36	1.08	0.74	0.46	0.16
**TG >95^th^ percentile**	0.59 (0.57–0.61)	0.59 (0.57–0.61)	54.0	93.3	56.9	12.5	39.6	35.8	70.3	78.1	1.26	1.06	0.81	0.58	0.15
**HDL-cholesterol <5^th^ percentile**	0.55 (0.53–0.57)	0.55 (0.53–0.57)	52.4	91.7	55.4	11.5	31.9	29.3	74.5	77.7	1.15	1.03	0.87	0.73	0.14
**Glucose** ≥**5.6 mmol/L**	0.50 (0.44–0.56)	0.50 (0.44–0.56)	51.3	87.8	53.4	10.4	3.8	3.4	96.8	96.0	1.08	0.97	0.94	1.2	0.03

AUROC, Area Under the Receiver Operating characteristic Curve; PPV, Positive Predictive Value; NPV, Negative Predictive Value; LR, Likelihood Ratio; CDC, Centers for Disease Control and Prevention; WHO, World Health Organization; BMI, Body Mass Index; BP, Blood Pressure; HDL, High Density Lipoprotein; CI, Confidence Interval.

**Table 4:** AUROC, sensitivity, specificity, PPV, NPV, LR^+^, LR^−^ and kappa of categories of severe obesity defined as BMI ≥1.2 times the 95^th^ percentile by CDC or WHO for predicting individual cardiometabolic risk.

	Severe obesity defined as BMI ≥1.2 times the 95^th^ percentile														
	AUROC (95%CI)		Sensitivity		Specificity		PPV		NPV		LR^+^		LR^−^		kappa
	CDC	WHO	CDC	WHO	CDC	WHO	CDC	WHO	CDC	WHO	CDC	WHO	CDC	WHO	
**SBP/DBP ≥95^th^ percentile**	0.61 (0.59–0.63)	0.62 (0.59–0.64)	71.5	86.8	40.9	22.8	38.6	36.9	73.4	76.9	1.20	1.13	0.71	0.56	0.64
**TG >95^th^ percentile**	0.58 (0.56–0.60)	0.58 (0.56–0.60)	71.3	87.1	40.8	23.0	38.6	37.1	73.1	77.3	1.20	1.13	0.71	0.56	0.64
**HDL-cholesterol <5^th^ percentile**	0.56 (0.54–0.58)	0.56 (0.54–0.58)	68.9	85.4	38.9	21.2	31.0	29.9	75.9	77.5	1.13	1.07	0.94	0.71	0.61
**Glucose ≥5.6 mmol/L**	0.52 (0.46–0.58)	0.52 (0.46–0.58)	60.9	73.9	36.6	19.3	3.3	3.2	96.3	95.4	0.96	0.91	1.05	1.37	0.21

AUROC, Area Under the Receiver Operating characteristic Curve; PPV, Positive Predictive Value; NPV, Negative Predictive Value; LR, Likelihood Ratio; CDC, Centers for Disease Control and Prevention; WHO, World Health Organization; BMI, Body Mass Index; BP, Blood Pressure; HDL, High Density Lipoprotein; CI, Confidence Interval.

**Figure 2:**
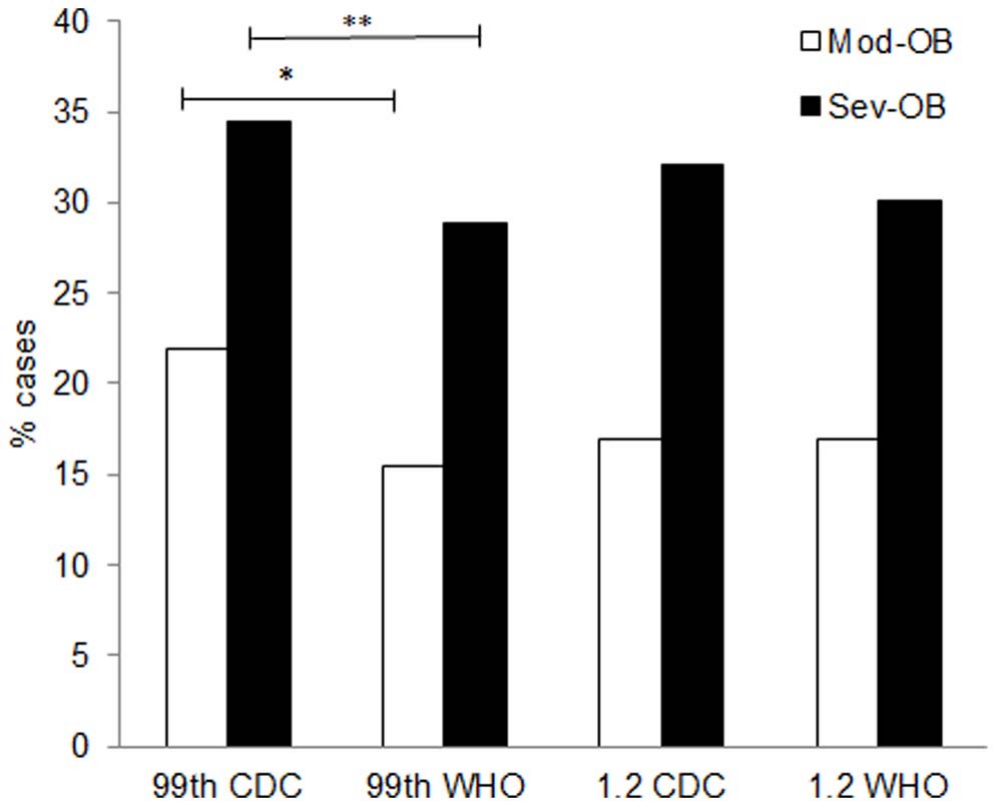
Relative frequency of clustered cardiometabolic risk factors in children with moderate (Mod-OB) or severe obesity (Sev-OB) defined according to BMI-for-age ≥99^th^ percentile or ≥1.2 times the 95^th^ percentile, by CDC or WHO.

As previously indicated in **Table 1**, 1637 children were classified as Mod-OB by CDC and 316 by WHO. The proportion of subjects with ≥2 cardiometabolic risk factors was higher in Sev-OB than Mod-OB independently of the classification system (p<0.0001) (**Figure 2**). Using the 99^th^ percentile, the frequency of Sev-OB subjects with ≥2 cardiometabolic risk factors was significantly higher with CDC than WHO (34.4 vs. 28.9%, p<0.0003), while no difference was found with the 1.2 times the 95^th^ percentile (32.1 vs. 30.1%, p = 0.147). Similar findings were found in groups classified as Mod-OB (**Figure 2**). **Tables 5** **and** **6** synthetize AUROC, sensitivity, specificity, PPV, NPV, LR^+^, LR^−^ and kappa coefficients of CDC and WHO defined categories of Sev-OB for predicting clustered cardiometabolic risk. Again, compared with WHO, the CDC 99^th^ percentile had lower sensitivity and NPV, but higher specificity and PPV in identifying children with clustered cardiometabolic risk factors (**Table 5**). Similar results were found in children stratified by gender or age. The discrepancies between CDC and WHO in sensitivity, specificity, PPV, and NPV were reduced using the 1.2 times the 95^th^ percentile (**Table 6**). Within the CDC system, the sensitivity in identifying patients with clustered cardiometabolic risk increased from 58.5% (99^th^ percentile) to 73.9% (1.2 times the 95^th^ percentile). Generally the definition based on CDC system had higher LR^+^ than the respective WHO system. For instance, a child with ≥2 cardiometabolic risk factors was 1.38 times more likely to have a BMI ≥99^th^ percentile according to CDC than it was a child with <2 cardiometabolic risk factors, while it was only 1.07 times more likely to have a BMI ≥99^th^ according to WHO than it was a child with <2 cardiometabolic risk factors. Using the BMI 99^th^ percentile, kappa coefficients indicated slight agreement between growth curves, with modestly higher values in boys than girls, and in younger than older children. Using the 1.2 times the 95^th^ percentile, kappa coefficients were greater than 0.60 in boys and in children ≤10 years, indicating substantial agreement between CDC and WHO growth curves.

**Table 5:** AUROC, sensitivity, specificity, PPV, NPV, LR^+^, LR^−^ and kappa of categories of severe obesity defined as BMI ≥99^th^ percentile by CDC or WHO for predicting clustered cardiometabolic risk in the total sample and in groups stratified by gender and age.

	Severe obesity defined as BMI ≥99^th^ percentile														
	AUROC (95%CI)		Sensitivity		Specificity		PPV		NPV		LR^+^		LR^−^		kappa
	CDC	WHO	CDC	WHO	CDC	WHO	CDC	WHO	CDC	WHO	CDC	WHO	CDC	WHO	
**Total**	0.61 (0.58–0.63)	0.607 (0.58–0.63)	58.5	94.2	57.6	12.3	34.4	28.9	78.6	84.9	1.38	1.07	0.72	0.47	0.15
**Males**	0.59 (0.56–0.62)	0.59 (0.56–0.61)	62.0	95.0	52.5	8.2	35.4	30.3	76.7	79.8	1.30	1.03	0.72	0.61	0.19
**Females**	0.63 (0.59–0.66)	0.62 (0.59–0.65)	54.2	93.2	62.7	16.3	33.0	27.4	80.1	87.7	1.45	1.11	0.73	0.74	0.11
**Age-class**															
≤**10 yrs**	0.55 (0.52–0.59)	0.55 (0.51–0.59)	71.2	98.4	36.9	4.7	31.2	29.4	76.1	88.4	1.13	1.03	0.78	0.78	0.35
≥**11yrs**	0.63 (0.61–0.66)	0.63 (0.61–0.66)	51.6	91.9	68.1	16.1	37.1	28.7	79.4	84.4	1.62	1.09	0.71	0.71	0.06

AUROC, Area Under the Receiver Operating characteristic Curve; PPV, Positive Predictive Value; NPV, Negative Predictive Value; LR, Likelihood Ratio; CDC, Centers for Disease Control and Prevention; WHO, World Health Organization; BMI, Body mass Index; CI, Confidence Interval.

**Table 6:** AUROC, sensitivity, specificity, PPV, NPV, LR^+^, LR^−^ and kappa of categories of severe obesity defined as BMI ≥1.2 times the 95^th^ percentile by CDC or WHO for predicting clustered cardiometabolic risk in the total sample and in groups stratified by gender and age.

	Severe obesity defined as BMI ≥1.2 times the 95^th^ percentile														
	AUROC (95%CI)		Sensitivity		Specificity		PPV		NPV		LR^+^		LR^−^		kappa
	CDC	WHO	CDC	WHO	CDC	WHO	CDC	WHO	CDC	WHO	CDC	WHO	CDC	WHO	
**Total**	0.62 (0.59–0.64)	0.62 (0.60–0.64)	73.9	88.1	40.7	22.5	32.1	30.1	80.4	83.3	1.25	1.18	0.64	0.53	0.61
**Males**	0.61 (0.58–0.64)	0.61 (0.58–0.64)	76.1	90.1	34.2	16.5	32.6	31.1	77.4	79.8	1.16	1.08	0.69	0.60	0.63
**Females**	0.62 (0.59–0.66)	0.63 (0.59–0.66)	71.1	85.8	47.0	28.3	31.3	28.9	82.7	85.4	1.34	1.19	0.61	0.50	0.59
**Age-class**															
**≤10 yrs**	0.58 (0.54–0.62)	0.59 (0.55–0.63)	80.8	92.9	26.3	14.3	30.6	30.4	77.3	83.3	1.09	1.08	0.73	0.49	0.72
**≥11yrs**	0.63 (0.61–0.66)	0.63 (0.61–0.66)	70.1	85.6	47.8	26.5	33.0	29.9	81.3	83.3	1.34	1.16	0.62	0.54	0.56

AUROC, Area Under the Receiver Operating characteristic Curve; PPV, Positive Predictive Value; NPV, Negative Predictive Value; LR, Likelihood Ratio; CDC, Centers for Disease Control and Prevention; WHO, World Health Organization; BMI, Body mass Index; CI, Confidence Interval.

## Discussion

As far as we are aware, our study compared for the first time the application of different classification methods of Sev-OB from two international systems to an overweight/obese population of children and adolescents. Depending on the classification system, a wide range of subjects was defined as Sev-OB. In particular, WHO standards provided significantly higher estimates of Sev-OB than CDC, mainly when using the 99^th^ percentile (+ 91.2%). The choice of one international system instead of the other and, moreover, of one criterion versus another may result in a wide variety of statistical definitions with clear implications of misclassification for screening policies and health resource planning [18]. The overestimation of the WHO system compared to CDC can be most likely ascribed to the “thinner” characteristics of reference populations used to draw WHO growth charts [19]. A recent interest from the pediatric research and medical communities has been turned to Sev-OB not only for its undesirable immediate consequences [14], [15], [20], but also for the relative resistance to current treatment approach, highlighting the importance of monitoring the transition from Mod-OB to Sev-OB. For instance, recent guidelines consider the 99^th^ percentile of BMI and other characteristics, such as age, health risks, and motivation of patient, as part of a treatment algorithm [21] to identify Sev-OB youths requiring more intensive interventions [22]. Behavioral treatment was successful for severely obese children aged 6–9 years [23], underlining the need of early identification and intervention. As our results pointed out, one third of the obese patients searching medical assistance were ≤10 years of age; almost all of them were classified as Sev-OB by WHO, using either the 99^th^ percentile (96%) or the 1.2 times the 95^th^ percentile of BMI (88%) when compared to CDC (65% and 76%, respectively). The very large prevalence of Sev-OB estimated by the WHO could be problematic, since it would practically imply that almost the entire population of obese children under 10 years of age searching medical assistance would require intensive treatment. The highest BMI and the worse cardiometabolic profile found in Sev-OB subjects identified by the 99^th^ percentile of CDC compared to those identified by the 99^th^ percentile of WHO, confirms that this latter system overestimates the frequency of Sev-OB.

It is worth noting how the adoption of different growth curves and diagnostic criteria seems to affect the identification of adverse cardiometabolic risk profile in Sev-OB children. As to the ability of categories of excess weight to identify patients with cardiometabolic risk, criteria based upon either the 99^th^ percentile or the 1.2 times the 95^th^ percentile of both CDC and WHO revealed that Sev-OB children had 54–93% higher prevalence of adverse cardiometabolic profile than Mod-OB children. This is in line with previous studies, which have demonstrated the deleterious effect of Sev-OB on health of children and adolescents [14], [15], [20].

While it was already established that the CDC 99^th^ percentile of BMI was a predictor of adverse health outcomes [24],[25], no study is available for the equivalent WHO cut-point. Recently, Kakinami et al. [26] compared the association between OW and OB as defined by CDC or WHO curves and the presence of cardiometabolic risk factors, demonstrating that, although the WHO classification showed higher sensitivity and lower specificity than CDC, the WHO curves showed no significant discriminatory advance over the CDC. The limited size of the obese sample did not allow further speculations regarding extreme obesity. In agreement with Kakinami et al. [26], we also found in a large sample of overweight/obese children that the sensitivity of CDC 99^th^ percentile in identifying Sev-OB children with single or clustered cardiometabolic risk factors was lower than WHO 99^th^ percentile, but yielded acceptable results (nearly 70%) when the 1.2 times the 95^th^ percentile CDC was used both in the total sample and in groups stratified by sex or age. On the contrary the specificity of CDC 99^th^ percentile was higher than WHO 99^th^ percentile, particularly in girls and youths ≥11 years. The agreement between methods in identifying single and clustered cardiometabolic risk factors greatly improved when the 1.2 times the 95^th^ percentile cutoff was used, in particular in children under 10 years of age. These data confirms that the choice of the 99^th^ percentile has yet some limitations, since it is for such reason imprecise with respect to lower percentiles. For instance, it has been underlined that the CDC growth chart data set is too sparse to construct smoothed percentiles beyond the 97^th^ percentile with adequate statistical reliability [27]. Probably, by applying the LMS method to growth data set from a large sample more representative of a general population (i.e. including severe obese patients), sensitivity and specificity to screen with the 99^th^ percentile Sev-OB patients at increased cardiometabolic risk would be significantly improved.

Our study may have some limitations. Firstly, overweight/obese subjects were recruited in pediatric obesity services, and may be not representative of the general population. Moreover, the investigation was limited to cardiometabolic risk factors, without considering mechanic or psychosocial complications associated with Sev-OB and along the whole spectrum of BMI. Lastly, the cross sectional design of the study does not allow to assess the ability of the BMI cutoffs to predict cardiometabolic outcomes in adulthood.

The decision as to which index of Sev-OB is used should depend on the application, therefore users of Sev-OB obesity definitions should be aware of the implications of the choice of index that they make. Overall, the 99^th^ percentile of WHO tends to greatly overestimate the prevalence of Sev-Ob, and this has considerable impact on the economic sustainability of health resource planning. As to clinical diagnostic applications, all classification methods had moderate/high accuracy (sensitivity) and poor precision (specificity) for the screening of CVD risk in children. Actually BMI is not the only factor influencing cardiometabolic risk. In any case, accuracy and precision improved when the cutoffs of 1.2 times the 95^th^ percentile of both CDC and WHO were employed. Interestingly, the very recent publication of new CDC growth charts defining a child's BMI as a “percentage of the 95^th^ percentile” [28] highlights the clinical interest towards this evaluation system, allowing clinicians to track and visualize BMI values in severely obese children.

In conclusion, we focused on the present-day clinical impact of moving from one set of growth curves to another and from one criterion to another in the screening of Sev-OB and adverse cardiometabolic profile in children and adolescents. Independently of the method used to define Sev-OB, the predictive value for cardiometabolic risk factors was low. However, considering the statistical limits of available percentiles at the extreme of BMI distribution, our results indicate that the 1.2 times the 95^th^ percentile of BMI of either CDC or WHO standards has a discriminatory advantage over the 99^th^ percentile for identifying Sev-OB children at increased cardiometabolic risk, particularly under 10 years of age. Moreover, this cutoff has the advantage to be used as a continuous variable, being suitable for practical reason or comparative studies. Considering the epidemics of extreme obesity in children, internationally accepted criteria for defining severe obesity are urgently needed.
